# Transcriptome Dynamics of BmN Cells During the Early Phase of *Bombyx mori* Nucleopolyhedrovirus Infection

**DOI:** 10.3390/insects17010080

**Published:** 2026-01-09

**Authors:** Xiong Wang, Fangyu Miao, Wei Wang, Jingchen Sun, Lunguang Yao

**Affiliations:** 1 Henan Provincial Engineering and Technology Center of Health Products for Livestock and Poultry, College of Life Science, Nanyang Normal University, Nanyang 473000, China; wangttx@outlook.com (X.W.); miaofy2025@163.com (F.M.); Wangwei10319@outlook.com (W.W.); 2 Guangdong Provincial Key Laboratory of Agro-Animal Genomics and Molecular Breeding, College of Animal Science, South China Agricultural University, Guangzhou 510642, China

**Keywords:** *Bombyx mori*, nuclear polyhedrosis virus, viral infection, transcriptome analysis

## Abstract

The silkworm, *Bombyx mori*, is an economically important insect severely impacted by *Bombyx mori* nucleopolyhedrovirus (BmNPV), a double-stranded DNA virus that causes substantial losses to the sericulture industry. To elucidate the mechanisms of BmNPV infection and replication, we performed transcriptome sequencing of BmN cells infected with a recombinant BmNPV at 12 and 24 h post infection (hpi). A total of 1136 differentially expressed genes (DEGs) were identified in the 12 hpi group, including 789 up-regulated and 347 down-regulated genes, while 5191 DEGs were detected at 24 hpi, including 2102 up-regulated and 3089 down-regulated genes. Functional annotation via GO and KEGG analyses highlighted the ECM–receptor interaction pathway as particularly significant. Furthermore, RT-qPCR results demonstrated that Map3k12 inhibits BmNPV replication. These findings lay the groundwork for further investigation into the molecular mechanisms of BmNPV infection and can be utilized for breeding dominant genes conferring resistance to nucleopolyhedrovirus in the silkworm.

## Simple Summary

*Bombyx mori* are one of the most economically significant model organisms, and their production is frequently severely threatened by *Bombyx mori* nucleopolyhedrovirus (BmNPV). In this study, we successfully generated recombinant BmNPV particles in vitro using a laboratory-established MultiBac system and infected the *B. mori* cell line BmN. Transcriptome data were collected at 12 hpi and 24 hpi. Analysis of the data revealed that the Map3k12 protein may inhibit BmNPV replication. This study not only provides data support for screening BmNPV receptors and identifying key proteins involved in viral replication, but also lays an important foundation for exploring BmNPV control strategies at the molecular level.

## 1. Introduction

The domestic silkworm, *Bombyx mori*, which belongs to the family Bombycidae within the order Lepidoptera, is a classic holometabolous insect [1,2]. Its developmental cycle sequentially progresses through four distinct stages—egg, larva, pupa, and adult—each exhibiting significant morphological and functional differences. As an insect of major economic importance, the silkworm plays a central role in global sericulture and serves as an irreplaceable foundation for the silk industry. For centuries, silk production has been a crucial pillar of agricultural economies in many regions, particularly in Asia, where it sustains livelihood security and provides employment for millions of people in rural areas [3,4]. Their well-defined genetic background, short life cycle, and ease of laboratory manipulation make *Bombyx mori* an ideal model for studying disease resistance mechanisms and virus–host interactions in Lepidoptera.

*Bombyx mori* nucleopolyhedrovirus (BmNPV), a significant member of the Baculoviridae family, possesses a double-stranded circular DNA genome. It extensively infects the midgut epithelial cells of the silkworm, causing substantial annual losses in the global silk industry [5,6]. The viral particles exhibit a structural and functional dichotomy: inclusion-derived viruses (ODVs) are embedded within a polyhedral matrix and mediate primary infection orally within the alkaline midgut; in contrast, budded viruses (BVs) are released from the basal membrane of infected cells to drive systemic infection through cell-to-cell transmission [7,8]. The infection cycle begins when silkworms ingest BmNPV-contaminated mulberry leaves. The viral polyhedra dissolve in the alkaline midgut, releasing ODVs [9], which then fuse with the midgut epithelial cell membrane [10]. Mediated by the envelope protein, they recognize specific receptors on the host cell membrane surface and enter the cell via endocytosis. Once inside the cell, the virus releases its nucleocapsid, which is transported to the nucleus guided by actin. Within the nucleus, the virus initiates gene transcription and assembles progeny virus nucleocapsids. Assembled nucleocapsids traverse the nuclear pore complex into the cytoplasm. Guided by the envelope protein, they bud off and acquire the host cell membrane as their viral envelope, ultimately forming mature progeny virus particles [11]. These progeny viruses utilize the envelope protein to recognize and infect additional host cells, repeating the infection cycle to enable intercellular transmission and secondary infections within the host.

Substantial progress has been made in understanding the resistance mechanisms of the silkworm to BmNPV, and numerous resistance-associated genes have been identified. For example, BmSOCS2 enhances resistance by regulating the JAK/STAT pathway [12], BmFerritin influences viral infection by modulating ROS levels, and BmRas3 suppresses BmNPV replication by activating the MAPK pathway [13]. However, key effector genes remain unidentified, particularly the receptor protein for BmNPV, which requires further investigation.

In this study, we first successfully constructed the budding-type BmNPV viral particle rBmBv-mCherry. Subsequently, we employed time series transcriptomics to systematically analyze the host response of *B. mori* cells during BmNPV infection. Specifically, BmN cells were inoculated with recombinant baculovirus, and samples were collected at 12 and 24 hpi for RNA sequencing analysis. Differential expression analysis identified a set of significantly up-regulated and down-regulated candidate genes. Further GO functional and KEGG pathway enrichment analyses revealed significant enrichment in the ECM–receptor interaction pathway. Moreover, RT-qPCR validation demonstrated that MAP3K12 inhibits BmNPV replication. Collectively, this study provides novel insights into the molecular mechanisms of baculovirus–host interactions and identifies potential molecular targets for developing BmNPV-resistant silkworm strains through molecular breeding.

## 2. Materials and Methods

### 2.1. Genes, Plasmids, Strains, and Cells

Plasmid pFBDM, plex4, monomeric Cherry fluorescent protein (mCherry), and *Escherichia coli* strains TOP10 and BmSw106-inv (which carry the baculovirus genome) were maintained in our laboratory [14]. The *Bombyx mori* ovarian cell line, BmN, was cultured in Grace’s insect cell culture medium supplemented with 10% fetal bovine serum (Gibco), 100 U/mL penicillin, and 100 μg/mL streptomycin (Gibco, Waltham, MA, USA).

### 2.2. Construction of Recombinant Baculovirus rBmNPV-mCherry

The mCherry gene was amplified using the primer pair in Table 1. The resulting PCR fragments and plasmid pFBDM were digested with restriction enzymes *SaI*I and *Sac*I (New England Biolabs, Ipswich, MA, USA) and ligated using T4 ligase (Fermentas, Waltham, MA, USA) at 16 °C. The recombinant vector pFBDM-mCherry was transformed into *E. coli* and subsequent DNA was obtained from cells. Subsequently, the recombinant *E. coli* strain rBmSw106-mCherry was obtained by transposition of the vector pFBDM-mCherry into *E. coli* strain BmSw106 via the Tn7 transposition site. Then, BmN cells were infected with the rBmSw106-mCherry strain to produce the recombinant baculovirus, rBmNPV-mCherry, which was typically harvested 3–5 days post infection.

### 2.3. Sample Preparation and RNA Sequencing

BmN cells were cultured in 24-well plates (Thermo, Waltham, MA, USA) and cultured in Grace’s medium at 28 °C. Upon 70–80% confluency, the cells were either infected with the recombinant baculovirus rBmNVP-mCherry (MOI = 1) or incubated with PBS as a mock uninfected control. Cells were then harvested at 12 and 24 hpi, with total RNA extracted using the NucleoZOL reagent kit (Macherey-Nagel, Düren, Germany). Each group had three replicate samples.

The purity of the extracted total RNA was assessed by measuring the A260/A280 ratio using a Nanodrop spectrophotometer (ThermoFisher Scientific, Waltham, MA, USA), and the quality and integrity (RIN) were assessed using an Agilent 2100 (Agilent Technologies, Santa Clara, CA, USA). Libraries of cDNA with the fragment size of 200 bp cDNA were constructed with the Hieff NGS Ultima Dual-mode mRNA Library Prep Kit (Yeasen, Shanghai, China). The samples were divided into twelve groups: BmN-12h-1, BmN-12h-2, BmN-12h-3 and BmN-12h-1-ck, BmN-12h-2-ck, BmN-12h-3-ck, BmN-24h-1, BmN-24h-2, BmN-24h-3 and BmN-24h-1-ck, BmN-24h-2-ck, and BmN-24h-3-ck (CK for control group). RNA sequencing was completed by Genedenovo Biotechnology Co., Ltd. (Guangzhou, China) using the sequencing platform Novaseq X plus (Illumina, San Diego, CA, USA).

### 2.4. Reads Filtering, Transcription Assembly, and Basic Annotation of Unigenes

Raw reads were processed using fastp V0.20.0 [15], with filtering criteria including the following: removal of reads containing adapter sequences, exclusion of reads with N-base proportion exceeding 10%, elimination of reads consisting entirely of A bases, and removal of low-quality reads (i.e., those with over 50% of bases having a quality score Q ≤ 20). Following these steps, high-quality clean reads were obtained for subsequent analysis. Clean reads were aligned to the reference genome of SilkDB3.0 (https://silkdb.bioinfotoolkits.net/ (accessed on 6 May 2023)) by HISAT2 V2.1.0 [16]. The reads were assembled into Unigenes using the StringTie V1.3.4 [17]. By aligning Unigene sequences to protein databases NR (NCBI non-redundant protein sequence), SwissProt (Swiss-Prot Protein Sequence Database), KEGG (Kyoto Encyclopedia of Genes and Genomes), and KOG (euKaryotic Orthologous Groups) via blastx (E-value < 0.00001), the protein with the highest sequence similarity to a given Unigene is identified, thereby providing functional annotation information for that Unigene.

### 2.5. Differential Expression Genes Analysis and Enrichment

Differentially expressed genes (DEGs) were identified using edgeR V3.22.3 [18]. Differentially expressed genes were defined as those with a false discovery rate (FDR) < 0.05 and | log_2_FC (Fold Change) | > 1. Subsequently, all selected DEGs were annotated against the Gene Ontology (GO) and the Kyoto Encyclopedia of Genes and Genomes (KEGG) pathway databases to systematically identify their potential biological functions and pathways. Building upon this, enrichment analysis was performed using the hypergeometric distribution test, with all annotated genes serving as background, to identify GO terms and KEGG pathways significantly enriched (Q-value < 0.05) within the DEGs.

### 2.6. The Vector plex4-Map3k12 Construction for Overexpression

The Map3k12 gene was amplified using the primers listed in Table 1, with cDNA derived from BmN cells as the template. The resulting PCR fragment was digested with the restriction enzymes *Kpn*I and *Not*I and subsequently cloned into the expression vector plex4, which is maintained in our laboratory, using T4 DNA ligase. Then, the overexpression vector plex4-Map3k12 was constructed successfully.

### 2.7. RNAi

Double-stranded RNA (dsRNA) was synthesized using the T7 RIBO MAX™ Express RNAi System kit (Promega, Madison, WI, USA), with the resulting product stored at −80 °C. Following mixing of the dsRNA with transfection reagent (Promega, USA), the mixture was added to BmN cells and incubated at 28 °C. At 24 h after 5 μg dsRNA transfection, the recombinant baculovirus BmBV-mCherry was introduced. Samples were collected at 12 and 24 hpi. BmN cells treated with dsEGFP served as the control group.

### 2.8. Reverse Transcription Quantitative Real-Time PCR (RT-qPCR)

Total RNA was extracted from BmN cells using the NucleoZOL reagent kit (Macherey-Nagel, Düren, Germany). Subsequently, cDNA was synthesized from the extracted RNA using a reverse transcription kit with gDNA eraser (Takara, Kusatsu, Japan). The RT-qPCR reaction mixture had a total volume of 20 μL, containing 0.8 μL of cDNA, 0.5 mM of each specific primer, and 10 μL of 2× TaqTM Universal SYBR Green Supermix (Bio-Rad, Hercules, CA, USA). Three technical replicates were performed for each sample. The amplification protocol was as follows: initial denaturation at 95 °C for 5 min, followed by 40 cycles of denaturation at 95 °C for 15 s and extension at 60 °C for 35 s. The relative quantification method (2^−ΔΔCt^) was used to evaluate the differential expression level, and the GAPDH gene of *B. mori* was used as the internal reference control to normalize the expression levels of all verified genes.

## 3. Results

### 3.1. Construction of Recombinant Baculovirus rBmBV-mCherry

**Figure 1:**
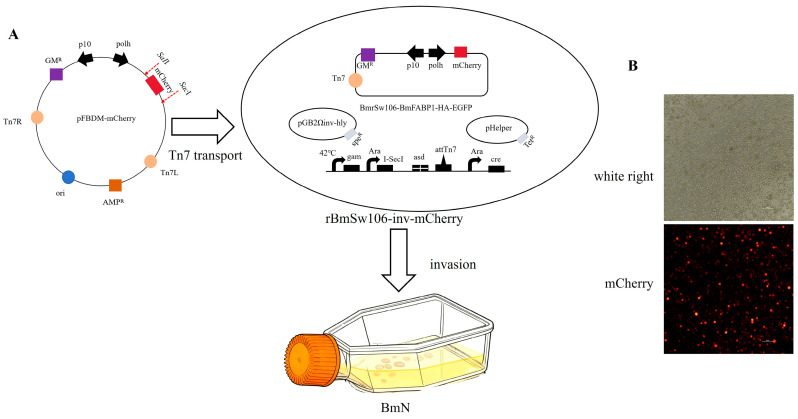
Construction and initial characterization of recombinant BmNPV. (**A**) Schematic of the construction strategy for rBmBV-mCherry. (**B**) Detection of primary recombinant baculovirus rBmBV-mCherry production by inverted fluorescence microscope (bar = 100 µm).

The constructed transfer vector pFBDM-mCherry was transferred into *E. coli* BmSw106-inv via the Tn7 transposon site. Positive colonies were screened on solid LB medium containing Kanamycin (Kan), Spectinomycin (Sep), Tetracycline (Tet), Gentamicin (Gm), and Diaminopimelic Acid (DAP). Subsequently, the successfully transposed recombinant *E. coli* strain rBmSw106-inv-mCherry was used to infect BmN cells. Finally, the recombinant baculovirus construct rBmBv-mCherry was obtained at 96 hpi (Figure 1).

### 3.2. RNA Sequencing Analysis

BmN cells were infected with the recombinant baculovirus rBmBv-mCherry, and cell samples were harvested at 12 and 24 hpi for subsequent transcriptome. A total of 638,229,020 raw reads were obtained from BmN cells samples. The removal of low-quality reads yielded 634,333,390 clean reads. The assessment of sequencing data quality revealed that the average Q20 and Q30 values were 97% and 93%, respectively, with a GC content ranging from 41% to 42%. Alignment with the silkworm reference genome was efficient in control samples, with mapping rates of 82–83%. In infected samples, however, the alignment rate dropped markedly from an average of 47% at 12 hpi to only 15% at 24 hpi (Table 2). This pronounced reduction in host genome-mapped reads likely reflects the increasing abundance of viral transcripts during infection, which were not alignable to the host reference genome.

### 3.3. Differental Expression Analysis of Genes

**Figure 2:**
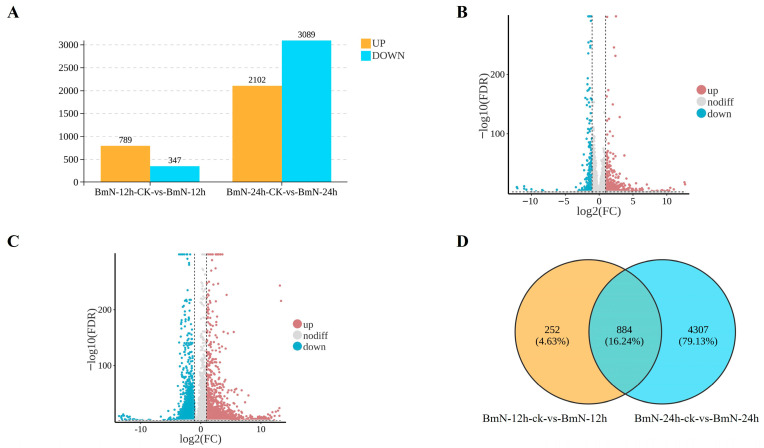
Transcriptional expressions of genes in non-infection and BmNPV infection at 12 hpi and 24 hpi. (**A**) DEGs at 12 hpi and 24 hpi. (**B**) Volcano plot of 12 hpi. (**C**) Volcano plot of 24 hpi. (**D**) The number of up- or down-regulated DEGs shown by Venn diagram.

A total of 1136 DEGs were identified in the 12 h treatment group, with 789 up-regulated and 347 down-regulated. In contrast, the 24 h treatment group yielded 5191 DEGs, including 2102 up-regulated genes and 3089 down-regulated genes (Figure 2A). These results were visualized using a volcano plot (Figure 2B,C). Further analysis using a Venn diagram revealed 884 DEGs common to both time points. Additionally, 252 DEGs were unique to the 12 h group, while 4307 DEGs were unique to the 24 h group (Figure 2D).

### 3.4. Enrichment Analysis of DEGs

To elucidate the dynamic functional changes in BmN following infection with BmNPV, GO enrichment analysis was performed on differentially expressed genes at 12 hpi and 24 hpi, covering three ontology categories: biological process (BP), molecular function (MF), and cellular component (CC).

**Figure 3:**
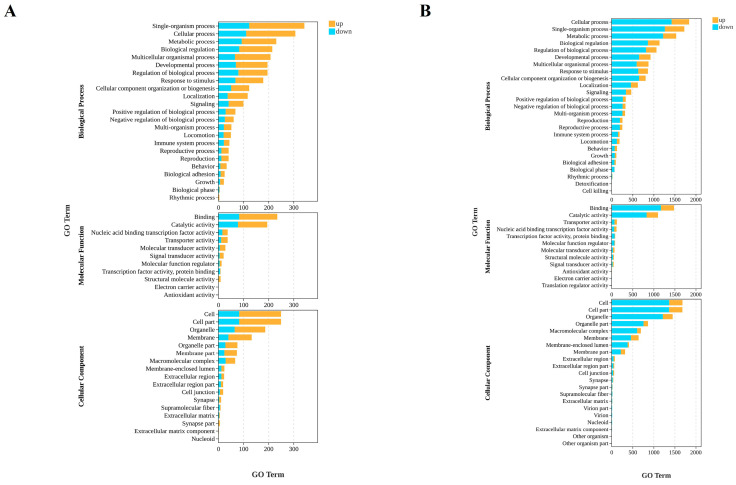
GO enrichment analysis of DEGs in mock-infected and BmNPV-infected cells. (**A**) 12 hpi. (**B**) 24 hpi.

At 12 hpi, DEGs were primarily enriched in BP related to fundamental cellular functions and regulatory mechanisms, such as single-organism processes and biological regulation. Within MF, core activities including binding and catalytic activity represented the dominant enriched terms. For CC, DEGs predominantly clustered in general subcellular structures including cells, organelles, and membranes. The enrichment levels at this time point were relatively low, suggesting an initial cellular response to BmNPV invasion (Figure 3A).

By 24 hpi, enrichment ranges and intensities across all biological categories significantly expanded. In BP, DEGs continued to accumulate in foundational categories like cellular processes and biological process regulation while further extending to more specialized pathways such as immune system processes and reproductive processes. Within MF, enrichment for binding activity and catalytic activity markedly increased, with new functional categories like structural molecular activity emerging. Regarding CC, DEGs began to accumulate in more complex cellular structures such as macromolecular complexes, membrane-bound compartments, and the extracellular matrix. Notably, compared with 12 hpi, the total number of DEGs increased substantially at 24 hpi, indicating that as the infection progressed, BmN functions underwent more profound and widespread disruption (Figure 3B).

**Figure 4:**
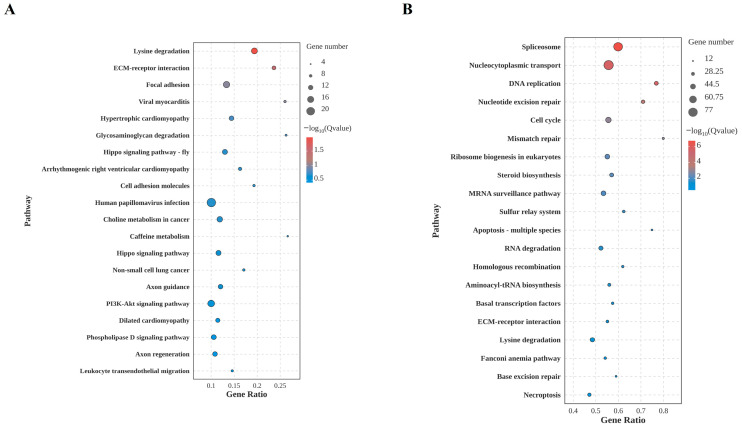
Top 20 pathways in the KEGG enrichment analysis of DEGs in mock-infected and BmNPV-infected cells. (**A**) 12 hpi. (**B**) 24 hpi.

In the KEGG enrichment analysis, the DEGs at 12 hpi were primarily enriched in pathways related to cell adhesion, basal metabolism, and certain signaling regulations. These included cell junction-associated pathways such as extracellular matrix–receptor interactions and focal adhesions; metabolic pathways like lysine degradation; and signaling pathways including Hippo and PI3K-Akt. These features suggest that pathway perturbations in BmN during early infection are concentrated at the physical interface and in fine-tuning basal metabolism. This likely represents that BmN initially adaptively regulates extracellular signaling and intracellular metabolic homeostasis during BmNPV invasion (Figure 4A).

By 24 hpi, the pathway enrichment profile of DEGs exhibited significant functional convergence and enhanced intensity. Core enriched pathways centered on BmN life processes, including transcriptional processing, DNA replication and repair, and cell cycle regulation. These included RNA/protein transport and processing pathways like spliceosome and nucleocytoplasmic transport, genomic metabolism pathways like DNA replication and nucleotide excision repair, and proliferation regulation pathways such as the cell cycle. Concurrently, stress-related pathways such as apoptosis and RNA degradation were enriched, indicating the BmN initiated a defense response (Figure 4B).

### 3.5. RT-qPCR Validation for Genes Expression Patterns

**Figure 5:**
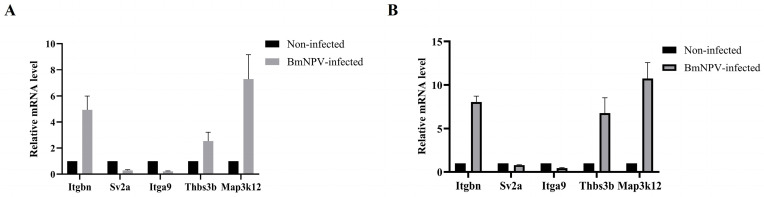
RT-qPCR analysis of some genes for the validation of RNA-Seq data. (**A**) 12 hpi. (**B**) 24 hpi.

We selected a subset of genes from the ECM–receptor interaction pathway, along with mitogen-activated protein kinase kinase kinase 12 (Map3k12), for RT-qPCR analysis. Itgbn (BMSK0001395), Thbs3b (BMSK0010961), and Map3k12 (BMSK0000069) were up-regulated at both 12 and 24 hpi. In contrast, Sv2a (BMSK0003084) and Itga9 (BMSK0006871) were down-regulated at the same time points (Table 3 and Table 4). The RT-qPCR results were consistent with those obtained from RNA-seq (Figure 5A,B).

### 3.6. The Role of Map3k12 in BmNPV Proliferation

**Figure 6:**
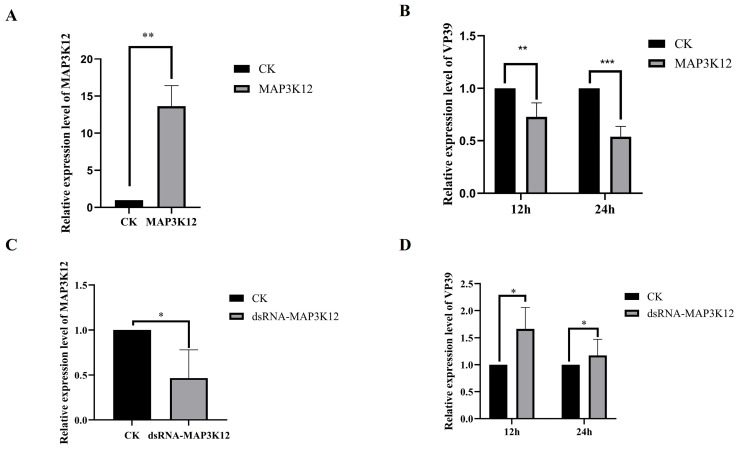
RT-qPCR analysis of the effect of Map3k12 on BmNPV replication. (**A**) The mRNA level of Map3k12 overexpression (** *p* < 0.01). (**B**) The mRNA level of VP39 on Map3k12-overexpressing cells (12 hpi, ** *p* < 0.01; 24 hpi, *** *p* < 0.001). (**C**) The mRNA level of Map3k12 down-regulated by dsRNA (* *p* < 0.05). (**D**) The mRNA level of VP39 on Map3k12 down-regulated cells (* *p* < 0.05).

To investigate the effect of Map3k12 on the proliferation of BmNPV at the mRNA level, we constructed the eukaryotic expression vector plex4-Map3k12 and transfected it into BmN cells. RT-qPCR analysis confirmed that Map3k12 mRNA expression was significantly up-regulated in transfected cells (Figure 6A). Subsequently, virus infection experiments were conducted on Map3k12-overexpressing cells, and the transcriptional levels of BmNPV nucleocapsid protein VP39 were detected via RT-qPCR. The results showed that compared with the control group, VP39 mRNA expression was significantly reduced at both 12 hpi and 24 hpi (Figure 6B).

Furthermore, dsRNA targeting Map3k12 was synthesized in vitro and transfected into BmN cells, down-regulating endogenous Map3k12 mRNA expression (Figure 6C). Subsequent viral infection experiments revealed that VP39 mRNA expression levels were significantly higher compared with the control group at 12 hpi and 24 hpi (Figure 6D). Collectively, these results indicate that Map3k12 exerts an inhibitory effect on BmNPV replication in BmN cells.

## 4. Discussion

BmNPV is one of the major viral pathogens of the silkworm and its cellular receptor remains unidentified. Transcriptome analysis in this study revealed significant enrichment of the ECM–receptor interaction pathway in the 12 hpi group, suggesting that this pathway may be involved in BmNPV viral entry. Integrins, as common cell surface receptors for ECM components, have been exploited by numerous enveloped and non-enveloped viruses for viral attachment [19]. Consequently, BmNPV may similarly enhance its initial attachment to BmN by binding to ECM components or receptors such as integrins, thereby facilitating subsequent membrane fusion and endocytosis. Furthermore, ECM–receptor interactions serve as critical hubs in cellular signal transduction. Upon binding to such receptors, viruses may activate or hijack downstream signaling pathways, such as MAPK, PI3K/Akt pathways, which are known to be activated during BmNPV infection and promote viral replication. Such signaling regulation may alter the intracellular environment, thereby facilitating viral entry, replication, and immune evasion [20].

**Figure 7:**
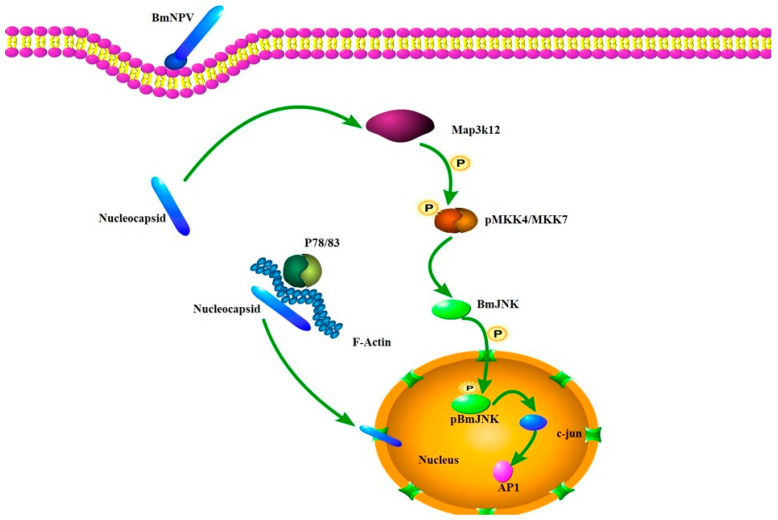
The hypothesis of Map3k12 influencing BmNPV replication through the JNK pathway.

Map3k12 is an upstream kinase in the MAPK signaling pathway, belonging to the MAP3K family, and participates in regulating various biological processes such as cellular stress responses, apoptosis, and differentiation [21]. In this study, we modulated the expression levels of Map3k12 through overexpression and RNA interference techniques. The RT-qPCR results indicated that Map3k12 exerts a certain inhibitory effect on Bv replication. As a kinase, Map3k12 can phosphorylate and activate MKK7/MKK4, thereby activating c-Jun N-terminal kinase (JNK) [22,23,24]. JNK plays a central role in multiple biological processes including cellular stress, apoptosis, inflammation, and immune responses. In viral replication studies, the time-dependent activation of the JNK pathway has been found to be crucial for viral particle production. BmNPV infection significantly activates the host ERK and JNK pathways within 12 hpi. Inhibiting these pathways at this stage substantially reduces the yield of polyhedrin ODVs and budding virus BV. Gene silencing experiments further revealed that knocking down BmErk/BmJnk inhibits the expression of viral late genes and viral assembly, potentially through mechanisms related to viral replication factory morphogenesis [25]. Additionally, it has been reported that BmFerHCH catalyzes Fe^2+^ conversion to Fe^3+^ via its ferrogenase activity, thereby limiting Fenton’s reactions and effectively suppressing ROS accumulation. Low ROS levels hinder JNK phosphorylation and nuclear translocation; conversely, BmFerHCH knockdown increases ROS accumulation, activating JNK phosphorylation and significantly inhibiting BmNPV proliferation [26]. Collectively, our functional data establish Map3K12 as a host inhibitor of BmNPV replication. Based on its known role as an upstream activator of the pro-apoptotic JNK pathway, we hypothesize that its antiviral effect may be mediated through the induction of apoptosis in infected cells, as summarized in the proposed model (Figure 7). However, this specific mechanistic link remains to be directly tested.

Of course, viral invasion and host defense represent a long-term process of coevolution [27,28,29]. Research also indicates that baculoviruses can encode apoptosis-inhibiting proteins (such as P35 and IAP) [30,31]. To successfully complete replication and enter the late production phase, viruses must effectively suppress premature apoptosis in host cells [32,33]. To validate the hypothesis presented in Figure 7, subsequent research will focus on elucidating the specific association between Map3k12 and BmNPV replication, particularly how Map3k12 influences the virus via the JNK pathway to exert its inhibitory effect.

## 5. Conclusions

In summary, this study obtained transcriptomic data of BmNPV at 12 hpi and 24 hpi. These data provide a proteomic reference for screening potential BmNPV receptors and identifying key proteins involved in viral replication, while also laying a foundation for subsequent research on the Map3k12 protein.

## Figures and Tables

**Table 1:** Primer used in this study.

Primer Name	Primer Sequence
mCherry-F	5′-AAGTCGACATGGTGAG-CAAGGGCGAGGAGCTGT-3′
mCherry-R	5′-AAGAGCTCTTACTTGTACAGCTCGTCCAT-3′
Map3k12-F	5′-AAGGTACCATGCTTTTTTTATCA-3′
Map3k12-R	5′-AAGCGGCCGCTTAGACGTGAGC-3′
Map3k12 for dsRNA synthesis-F	5′-GTAATACGACTCAC-TATAGGGAGCGGCAACCTTA-GAGGTGAAATGG-3′
Map3k12 for dsRNA synthesis-R	5′-GTAATACGACTCACTATAGGGCGGA-GACTTACCGTCCGACG-3′
EGFP for dsRNA synthesis-F	5′-GGATCCTAATACGACTCACTATAGGATGGTGAGCAAGGGC-3′
EGFP for dsRNA synthesis-R	5′-GGATCCTAATACGACTCACTATAGGTTACTTGTACAGCTCGTC-3′
Map3k12 for RT-qPCR-F	5′-GGGAAGTGCCATTTGAG-3′
Map3k12 for RT-qPCR-R	5′-CTATGTTGTCGTGGTTGAGT-3′
VP39 for RT-qPCR-F	5′-CTAATGCCCGTGGGTATGG-3′
VP39 for RT-qPCR-R	5′-TTGATGAGGTGGCTGTTGC-3′
GAPDH for RT-qPCR-F	5′-CATTCCGCGTCCCCTGTTGCTAAT-3′
GAPDH for RT-qPCR-R	5′-GCTGCCTCCTTGACCTTTTGC-3′

**Table 2:** Overview of the sequencing reads.

Sample	Raw Data	Clean Reads	Q20 (%)	Q30 (%)	GC (%)	Total Mapped (%)
BmN-12h-1	52,801,132	52,491,010	97.53	92.91	42.20	46.97
BmN-12h-2	50,967,124	50,628,832	97.57	92.90	42.17	47.40
BmN-12h-3	49,408,250	49,114,190	97.83	93.51	42.11	48.12
BmN-12h-1-ck	59,107,832	58,728,882	97.76	93.27	41.84	82.83
BmN-12h-2-ck	51,707,854	51,382,778	97.56	92.94	41.91	82.48
BmN-12h-3-ck	54,139,170	53,794,204	97.74	93.37	41.73	82.01
BmN-24h-1	39,745,074	39,516,804	96.90	91.49	42.29	15.56
BmN-24h-2	71,026,758	70,625,640	97.57	92.94	42.23	15.60
BmN-24h-3	65,696,490	65,337,122	97.89	93.61	41.95	15.27
BmN-24h-1-ck	57,915,664	57,557,250	97.45	92.69	42.07	82.99
BmN-24h-2-ck	36,151,820	35,909,688	98.06	94.19	42.47	83.87
BmN-24h-3-ck	49,561,852	49,246,990	97.92	93.83	42.82	83.99

**Table 3:** RNA-Seq results for selected DEGs at 12 hpi.

DEGs	log2Fold Change	Adjust *p* Value	Regulated
Itgbn	1.82	2.32 × 10^−4^	up
Sv2a	−1.26	2.20 × 10^−88^	down
Itga9	−1.70	9.68 × 10^−57^	down
Thbs3b	2.57	2.78 × 10^−5^	up
Map3k12	5.04	5.21 × 10^−5^	up

**Table 4:** RNA-Seq results for selected DEGs at 24 hpi.

DEGs	log2Fold Change	Adjust *p* Value	Regulated
Itgbn	1.68	0.0002	up
Sv2a	−2.51	5.72 × 10^−82^	down
Itga9	−3.25	7.12 × 10^−45^	down
Thbs3b	1.59	0.0007	up
Map3k12	3.31	1.14 × 10^−8^	up

## Data Availability

The original contributions presented in this study are included in the article/Supplementary Material. Further inquiries can be directed to the corresponding authors.

## Supplementary Materials

The following supporting information can be downloaded at: https://www.mdpi.com/article/10.3390/insects17010080/s1, Table S1: DEGs at 12 hpi; Table S2: DEGs at 24 hpi.
